# Photon and Proton irradiation in Patient-derived, Three-Dimensional Soft Tissue Sarcoma Models

**DOI:** 10.1186/s12885-023-11013-y

**Published:** 2023-06-22

**Authors:** Siyer Roohani, Jürgen Loskutov, Jens Heufelder, Felix Ehret, Lena Wedeken, Manuela Regenbrecht, Rica Sauer, Daniel Zips, Andrea Denker, Antonia M. Joussen, Christian R. A. Regenbrecht, David Kaul

**Affiliations:** 1 Charité – Universitätsmedizin Berlin, corporate member of Freie Universität Berlin and Humboldt-Universität zu Berlin, Department of Radiation Oncology, Augustenburger Platz 1, 13353 Berlin, Germany; 2 Charité – Universitätsmedizin Berlin, German Cancer Consortium (DKTK), partner site Berlin, and German Cancer Research Center (DKFZ), 69120 Berlin, Heidelberg, Germany; 3 CELLphenomics GmbH, Robert-Rössle-Str. 10, 13125 Berlin, Germany; 4 Charité – Universitätsmedizin Berlin, corporate member of Freie Universität Berlin and Humboldt-Universität zu Berlin, BerlinProtonen am Helmholtz-Zentrum Berlin, 14109 Berlin, Germany; 5 Charité – Universitätsmedizin Berlin, corporate member of Freie Universität Berlin and Humboldt-Universität zu Berlin, Department of Ophthalmology, 12200 Berlin, Germany; 6 Berlin Institute of Health at Charité – Universitätsmedizin Berlin, Charitéplatz 1, 10117 Berlin, Germany; 7 Helios Klinikum Berlin-Buch, Schwanebecker Chaussee 50, 13125 Berlin, Germany; 8 ASC Oncology GmbH, Robert-Rössle-Str. 10, 13125 Berlin, Germany; 9 Institute of Pathology, Helios Klinikum Emil von Behring, Walterhöferstr. 11, 14165 Berlin, Germany; 10 Helmholtz-Zentrum Berlin für Materialien und Energie, 14109 Berlin, Germany; 11 Institut für Pathologie, Universitätsmedizin Göttingen, Robert-Koch-Straße 40, 37075 Göttingen, Germany

**Keywords:** Soft tissue sarcoma, Sarcoma, 3D cell culture, Organoid, Patient-derived, Radiotherapy, Proton, Photon

## Abstract

**Background:**

Despite their heterogeneity, the current standard preoperative radiotherapy regimen for localized high-grade soft tissue sarcoma (STS) follows a one fits all approach for all STS subtypes. Sarcoma patient-derived three-dimensional cell culture models represent an innovative tool to overcome challenges in clinical research enabling reproducible subtype-specific research on STS. In this pilot study, we present our methodology and preliminary results using STS patient-derived 3D cell cultures that were exposed to different doses of photon and proton radiation. Our aim was: (i) to establish a reproducible method for irradiation of STS patient-derived 3D cell cultures and (ii) to explore the differences in tumor cell viability of two different STS subtypes exposed to increasing doses of photon and proton radiation at different time points.

**Methods:**

Two patient-derived cell cultures of untreated localized high-grade STS (an undifferentiated pleomorphic sarcoma (UPS) and a pleomorphic liposarcoma (PLS)) were exposed to a single fraction of photon or proton irradiation using doses of 0 Gy (sham irradiation), 2 Gy, 4 Gy, 8 Gy and 16 Gy. Cell viability was measured and compared to sham irradiation at two different time points (four and eight days after irradiation).

**Results:**

The proportion of viable tumor cells four days after photon irradiation for UPS vs. PLS were significantly different with 85% vs. 65% (4 Gy), 80% vs. 50% (8 Gy) and 70% vs. 35% (16 Gy). Proton irradiation led to similar diverging viability curves between UPS vs. PLS four days after irradiation with 90% vs. 75% (4 Gy), 85% vs. 45% (8 Gy) and 80% vs. 35% (16 Gy). Photon and proton radiation displayed only minor differences in cell-killing properties within each cell culture (UPS and PLS). The cell-killing effect of radiation sustained at eight days after irradiation in both cell cultures.

**Conclusions:**

Pronounced differences in radiosensitivity are evident among UPS and PLS 3D patient-derived sarcoma cell cultures which may reflect the clinical heterogeneity. Photon and proton radiation showed similar dose-dependent cell-killing effectiveness in both 3D cell cultures. Patient-derived 3D STS cell cultures may represent a valuable tool to enable translational studies towards individualized subtype-specific radiotherapy in patients with STS.

**Supplementary Information:**

The online version contains supplementary material available at 10.1186/s12885-023-11013-y.

## Introduction

Soft tissue sarcomas (STS) are a heterogeneous group of rare malignant tumors with more than 70 subtypes listed in the current World Health Organization (WHO) classification [1]. Despite their heterogeneity, guidelines still recommend the same radiotherapy (RT) regimen for all localized high-grade STS subtypes [2–7]. Standard therapy comprises preoperative RT in daily fractions of 1.8-2.0 Gy to a total dose of 50-50.4 Gy followed by wide resection [2, 4, 5, 8, 9]. The low incidence and diversity of STS make large clinical trials and subtype-specific clinical research particularly challenging [10]. Patient-derived 3D cell cultures (human organoids) have become a valuable tool in the study of human diseases, complementing and in some cases replacing animal studies [11–13]. In oncology, 3D cell cultures have been successfully used to study tumor development and progression and to test drug sensitivity [14–20]. Sarcoma patient-derived 3D cell cultures (PD3D) represent an innovative tool to overcome challenges in clinical research and to conduct reproducible subtype-specific analyses on STS [21–24]. Recently, Haas et al. used 2D cell lines to test subtype-specific radiosensitivity of STS [25]. The 14 well-characterized sarcoma cell lines showed striking differences in radiosensitivity after a single dose of photon irradiation (2–8 Gy). These findings, along with other preclinical data from 2D STS cell lines, suggest significant subtype-specific differences in radiosensitivity [25, 26]. However, 2D cell lines have important limitations. Forcing cells to grow on a 2D culture dish changes cellular morphology which leads to altered gene and protein expression and changes in cellular behavior compared to the tissue of origin [21, 27–30]. These limitations are partially overcome by PD3D that have a microenvironment similar to that of the donor tissue including the spatial organization, extracellular matrix, nutrient and oxygen gradients that allow tumor cells to have natural cell-cell and cell-matrix interactions [13, 21, 31]. Three-dimensional cell cultures allow the growth of cells in their natural shape and show greater genetical and phenotypical similarity to the tumors in vivo which also translates into improved predictability of the effects of ionizing radiation [32, 33]. Moreover, differences in hypoxia and cell radiosensitivity between tumor core and border can be visually assessed in 3D cell cultures as well [34]. Three-dimensional cell culture models can also be used for multi-omics analyses and single cell sequencing to further individualize tumor treatment (e.g., by drug sensitivity screenings, finding target mutations etc.), which may support clinical decision-making [22, 35–39]. To the best of our knowledge, there are no data on subtype-specific responses to radiation in STS 3D cell cultures yet. In this pilot study, we present our methodology and preliminary results using STS PD3D that were exposed to increasing doses of photon and proton radiation. We measured tumor cell viability at two different time points after irradiation. Our aim was: (i) to establish a reproducible method for irradiation of STS PD3D and (ii) to explore the differences in tumor cell viability of two different STS subtypes exposed to increasing doses of photon or proton radiation at two different measurement time points.

## Methods

### Patient-derived 3D STS cell culture

STS PD3D were prepared as previously described [40]. Briefly summarized, fresh surgical specimens underwent several steps of mechano-chemical dissociation. Subsequently, cell aggregates were seeded into 24-well plates (Corning, Amsterdam, Netherlands) in matrix-like scaffolds and allowed to grow until they started forming colonies. After harvesting, the cells underwent pathological examination to confirm origin and diagnosis. Culture conditions were similar to previously published projects with minor modifications [41]. Specifically, matrix-like scaffold was supplemented with collagen I (Corning, Amsterdam, Netherlands) and culture media was supplemented with PDGF-BB (Shenandoah-Biotechnology, Warminster, PA, USA).

In this study, two STS PD3D were used. Both were derived from previously untreated high-grade STS (G3 according to the Fédération Nationale des Centres de Lutte Contre le Cancer classification) [42]. One model (Sarc-P-53) is derived from a patient with a histopathologically diagnosed G3 undifferentiated pleomorphic sarcoma (UPS) who underwent preoperative radiochemotherapy with 1.8 to 50.4 Gy while the other model (Sarc-P-117) is derived from a patient with a G3 pleomorphic liposarcoma (PLS) who did not receive RT. To correctly characterize the tumor models, hematoxylin and eosin (H&E) and immunohistochemistry (IHC) staining were performed in the original tumor specimen and the 3D cell culture according to standard clinical protocols and reviewed by pathologists (supplementary Figs. 1 and 2). In preliminary experiments, the growth kinetics of the PD3D models with varying number of cultured tumor cells and culture media were analyzed to ensure irradiations were standardized and performed during the exponential growth phase to avoid overgrowth with subsequent necrosis of the core due to media exhaustion and oxygen gradients. If the control group was reaching confluency and thereby exiting the exponential growth phase, the results were not considered valid for analysis.

The experimental setup was arranged as previously described [18]. Sarcoma PD3D were mechanically dissociated to a single cell suspension. Single cells were mixed with a matrix-like scaffold at density 300–500 cells/µL and plated as 10µL domes in 24-well plates (Corning, Amsterdam, Netherlands). After polymerization of the scaffold, culture media supplemented with 10µM of Y27632 (MedChemExpress, Sollentuna, Sweden) was added and domes were gently detached from the plate. After four days in culture, domes were harvested and transported to the radiation facility. In each experiment, four domes were left in culture media at the cell culture laboratory to control for the effects of transportation. After irradiation, domes were transported back to the cell culture laboratory, placed back in 24-well plates and media was replaced by fresh culture media. After four or eight days in culture, PD3D were imaged by Leica DM IL LED Fluo microscope (Leica Mycrosystems, Wetzlar, Germany) and viability was evaluated using the CellTiter-Glo assay (Promega, Madison, WI, USA). The intensity of luminescence was measured using the SpectraMax i3x plate reader (Molecular Devices, San Jose, California, USA) 30 min after the addition of the reagent.

### Photon irradiation

Samples were transported in 1.5 ml Eppendorf tubes at room temperature from the cell culture laboratory to the linear accelerator facility. To ensure a homogeneous dose accumulation effect, the samples were placed in a plexiglass block with drill holes and a plexiglass cover plate designed for holding the samples during irradiation (supplementary Fig. 3). The holder was equipped with a dosimeter for internal dose control. Computed tomography scans of the plexiglass holder carrying the samples were taken for radiation planning (supplementary Fig. 4). Two-field three-dimensional conformal RT (anterior-posterior 0°, posterior-anterior 180°) were applied. The source-isocenter distance was 100 cm. Six MV photon radiation with dose rates up to 6 Gy/min were used. Each group of samples was irradiated with a single dose of either 0 Gy (sham irradiation), 2 Gy, 4 Gy, 8 Gy or 16 Gy as confirmed by dosimetric measurements during irradiation. In total, the time for irradiation of samples did not exceed 15 min. After irradiation, the samples were transported back to the cell culture laboratory.

### Proton irradiation

**Fig. 1:**
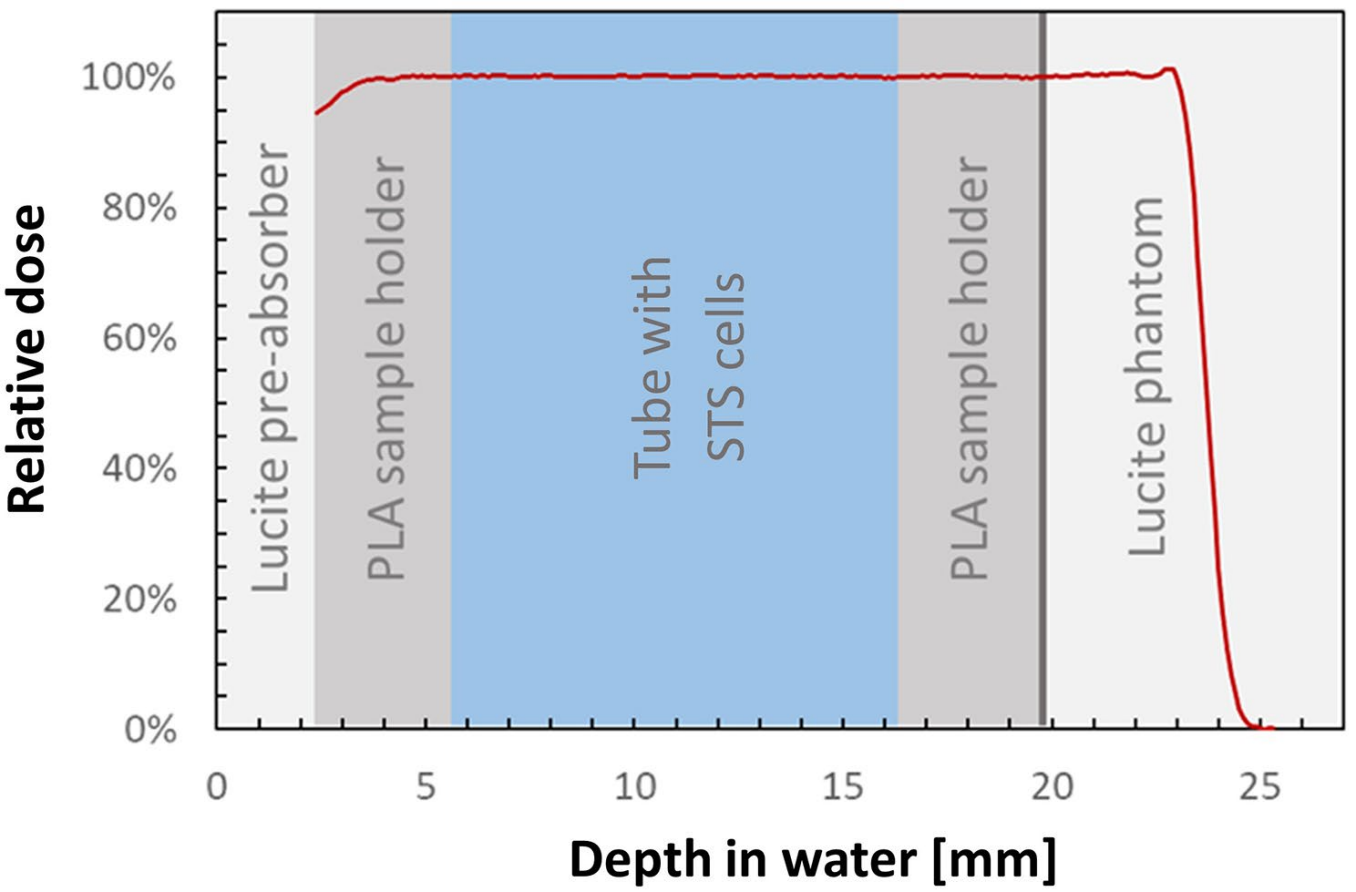
Depth dose curve of the spread out proton Bragg peak. After the Lucite pre-absorber (light grey) the beam enters the measurement chamber. The depth dose curve remains steady as it passes through the polylactide sample holder (grey), the Eppendorf tube with soft tissue sarcoma cells (light blue), reaches the exit dosimetry (vertical dark grey line at 19.8 mm depth) and displays a steep decline in the Lucite phantom (light grey).

The proton irradiation was performed at the ocular beam line of the Helmholtz-Zentrum Berlin für Materialien und Energie [43]. Standard 1.5ml Eppendorf tubes were used for sample transport and irradiation at room temperature. The following experimental setup of the proton beam line was used: Due to the small field size of the ocular beam line, only one Eppendorf tube was irradiated at a time, using one small circular field of 25 mm diameter. A 68 MeV proton beam was turned into a spread out Bragg peak with a water equivalent range of 23.7 mm and a modulation of 22.0 mm for irradiation. The tip of the tube containing the samples were positioned in the center of the irradiation field in 11.0 mm water equivalent depth. For adequate positioning, a special phantom consisting of a 2 mm pre-absorber Lucite plate with a 3D printed polylactide (PLA) sample holder of 17.0 mm was used. Distal to the sample holder, a Markus ion chamber (M23343-4795, PTW-Freiburg, Freiburg, Germany) was positioned in 19.8 mm water equivalent depth to measure the exit dose. Due to the physical characteristics of the spread out Bragg peak, the sample dose and the exit dose were the same with an error of ± 1% (Fig. 1). A dose rate of 3.9–4.1 Gy/min was used to apply the following doses based on exit dosimetry: 0 Gy (sham irradiation), 2 Gy, 4 Gy, 8 Gy, 16 Gy. The entire irradiation procedure including set-up and calibration took about 50 min. After irradiation, the samples were transported back to the cell culture laboratory.

### Statistical analysis and graphical representation

Statistical analysis was performed using GraphPad Prism v.9.3.1 (GraphPad Software, San Diego, CA, USA). For evaluation of the transportation effect, the unpaired two-tailed t-test was used. In all other cases, two-way ANOVA with Sidak’s multiple comparison for comparison of means between two correspondent groups (Figs. 5  and supplementary Figs. 5 and 6) or Dunnett’s multiple comparisons test for comparison of means to the control mean (Figs. 3 and 4) were used. Viability was expressed in percentage relative to sham irradiation (0 Gy). Bar charts were used to display results on the transportation effect (Fig. 5). Dose-response measurements were displayed for the different experimental set ups (Figs. 3, 4 and 5 and supplementary Figs. 5 and 6).

## Results

### Effect of transportation on cell viability

**Fig. 2:**
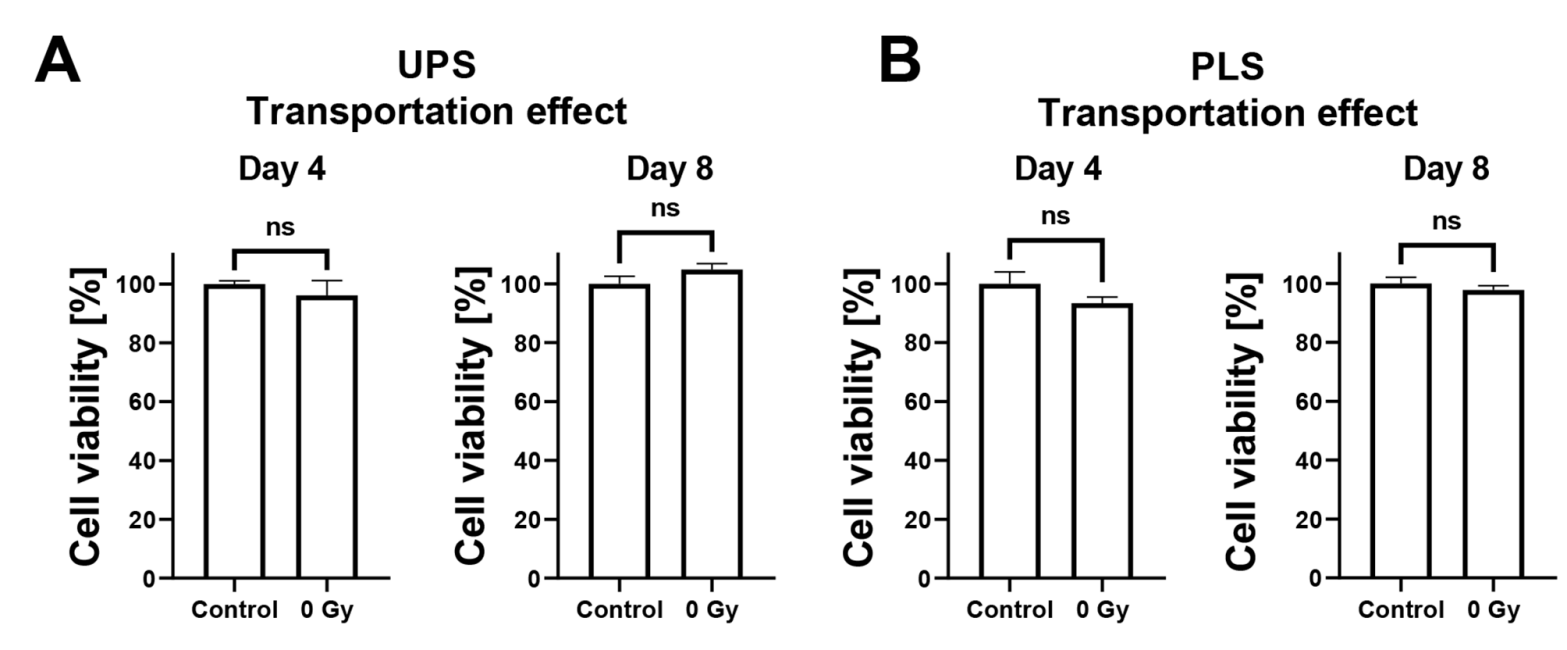
Effect of transportation on PD3D viability. Viability of UPS cell culture (**A**) and PLS cell culture (**B**) samples retained in the cell culture laboratory (Control) vs. sham irradiation (0 Gy) after four or eight days of incubation. Mean ± standard error of the mean; ns, not significant; 3 independent experiments with 4 technical replicates in each.

To determine the effect of transportation from the cell culture laboratory to the radiation facilities on cell viability (approximately 30 minutes of transport), we compared control samples that remained in the laboratory to transported samples receiving sham irradiation (0 Gy). Irradiations were conducted at the Department of Radiation Oncology at Charité - Universitätsmedizin Berlin (photon irradiation) and Helmholtz-Zentrum Berlin (proton irradiation) according to the same protocol. No significant differences were found among samples in both PD3D (UPS and PLS) on day 4 or day 8, respectively (Fig. 2). Therefore, the viability of the 0 Gy group (sham irradiation) was set as 100% in all subsequent calculations.

### Kinetics and dose-response of tumor cell viability after irradiation

**Fig. 3:**
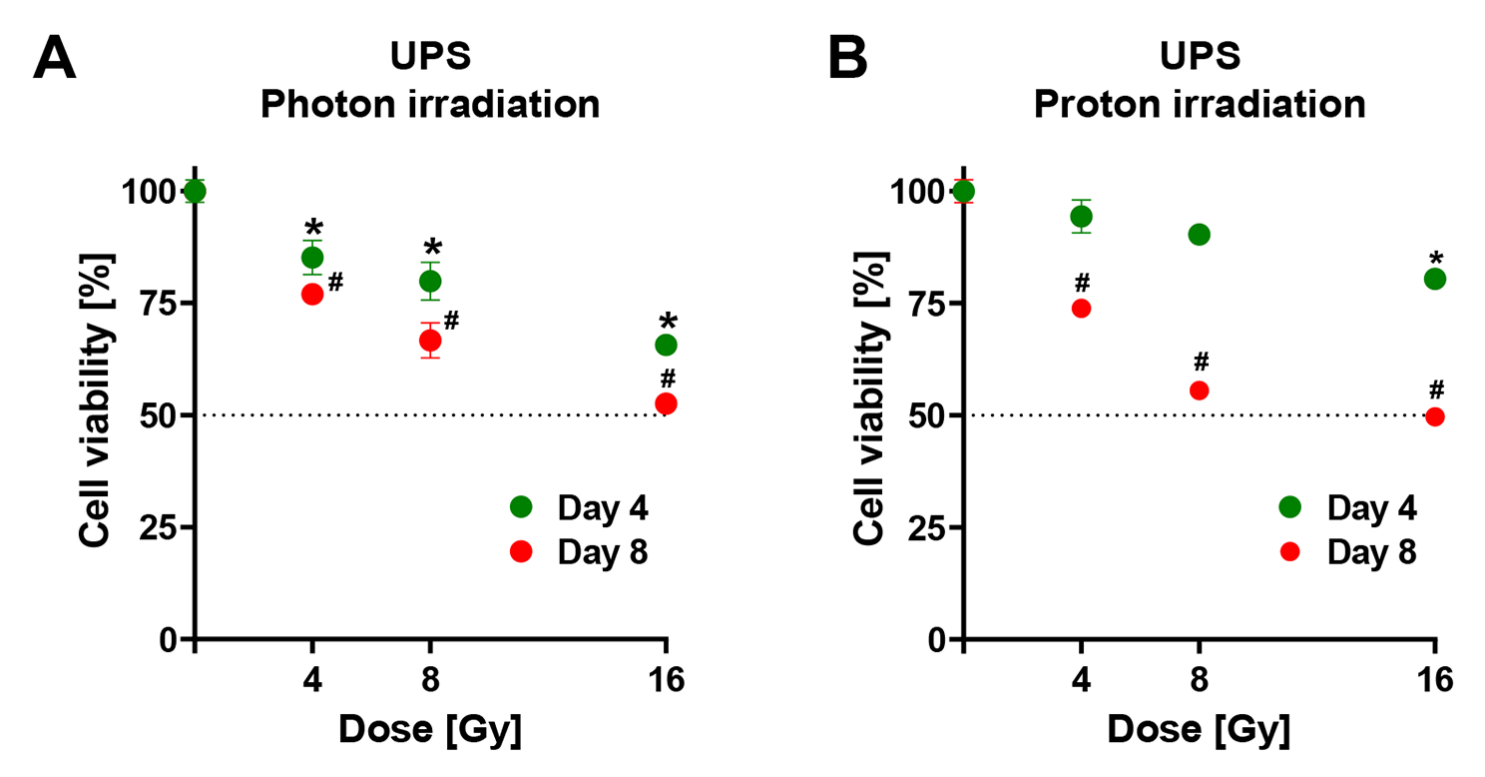
Dose response and kinetics of cancer cell viability following photon and proton irradiation of UPS (Sarc-P-53). Viability of UPS cell culture after four and eight days of incubation following increasing dosages of photon (**A**) or proton (**B**) irradiation. Mean ± standard error of the mean; *, p < 0.05 vs. sham irradiation (0 Gy) after four days; #, p < 0.05 vs. sham irradiation (0 Gy) after eight days; at least 1 experiment with 4 technical replicates in each.

In the photon irradiation group four days after irradiation, the number of viable UPS tumor cells were significantly reduced in a dose-dependent manner compared to sham irradiation (Fig. 3A). The effect increased at eight days with almost 50% of cell death when 16 Gy were applied. In the proton irradiation group, doses of up to 8 Gy did not cause a significant reduction of cell viability compared to sham irradiation, although an almost linear dose-dependent decrease was visible (Fig. 3B). Significant cell death levels of approximately 20% were seen four days after irradiation seen when 16 Gy were applied. Eight days after proton irradiation, the level of cell death increased in all dose-groups and reached approximately 50% in the 16 Gy group.

**Fig. 4:**
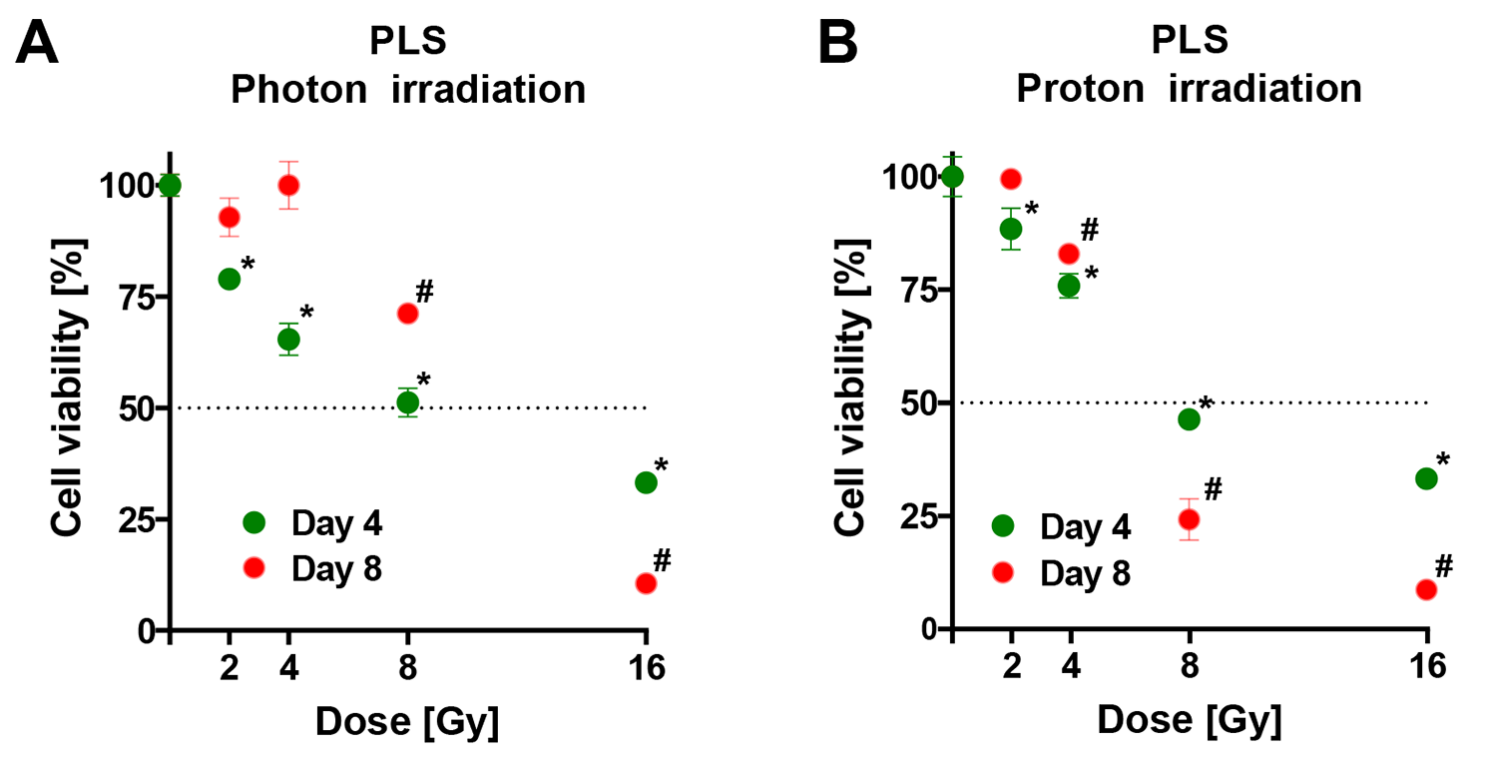
Dose response and kinetics of cancer cell viability following photon and proton irradiation of PLS (Sarc-P-117). Viability of PLS cell culture after four and eight days of incubation following increasing dosages of photon (**A**) or proton (**B**) irradiation. Mean ± standard error of the mean; empty circles, cells overgrown; *, p < 0.05 vs. sham irradiation (0 Gy) after four days; #, p < 0.05 vs. sham irradiation (0 Gy) after eight days; at least 1 experiment with 4 technical replicates in each.

The PLS tumor cell viability substantially decreased with increasing photon radiation doses at day 4 of measurement (Fig. 4A). In the 8 Gy group, 50% of cells were eradicated at four days. In the 16 Gy group 65% of cells were eliminated at four days. Eight days after irradiation, no significant differences were visible between the 2 and 4 Gy group compared to the sham irradiation group. However, 8 and 16 Gy led to a rapid decline with approximately 30% cell death in the 8 Gy group and 85% cell death in the 16 Gy group. Unfortunately, tumor cells in the PLS 0 Gy group were overgrown and confluent after 8 days which likely affected cell viability measurements. The proton irradiation groups showed a significant and dose-dependent decline in viability after four days reaching levels above 50% cell death in the 8 Gy group and around 65% in the 16 Gy group (Fig. 4B). Proton irradiation groups displayed a sigmoid shape with a plateau at 2 Gy and a subsequent rapid decline at 4 Gy (85%), 8 Gy (25%) and a minimum at 16 Gy (15%) in the eight days analysis. Tumor cells in 0 Gy group were overgrown and confluent after eight days.

### Effect of photon vs. proton radiation on tumor cell viability

In the UPS cell culture group, no significant differences in cell viability were visible between photon and proton irradiation after four days (supplementary Fig. 5A). Eight days after irradiation, there was a slightly higher cell-killing effect of proton irradiation visible in the 8 Gy group (supplementary Fig. 5B). Both showed dose dependent decreases in cell viability reaching approximately 50% in the 16 Gy groups after eight days.

Different kinetics were evident in the PLS cell culture. After four days, the cell-killing effect gradually increased with higher doses of irradiation reaching almost 65% of eradicated cells in the 16 Gy groups (supplementary Fig. 6A). Notably, there were no significant differences among photon or proton irradiation. At day eight, both groups showed a plateau in low doses and a rapid decline reaching a bottom plateau at 16 Gy (90% eliminated cells, supplementary Fig. 6B). In the lower doses (4 Gy, 8 Gy), photon irradiation displays less cell-killing properties than proton irradiation, however both measurement points converge at 16 Gy. Both cell cultures were overgrown at day eight.

### Differences in radiosensitivity between UPS and PLS PD3D

**Fig. 5:**
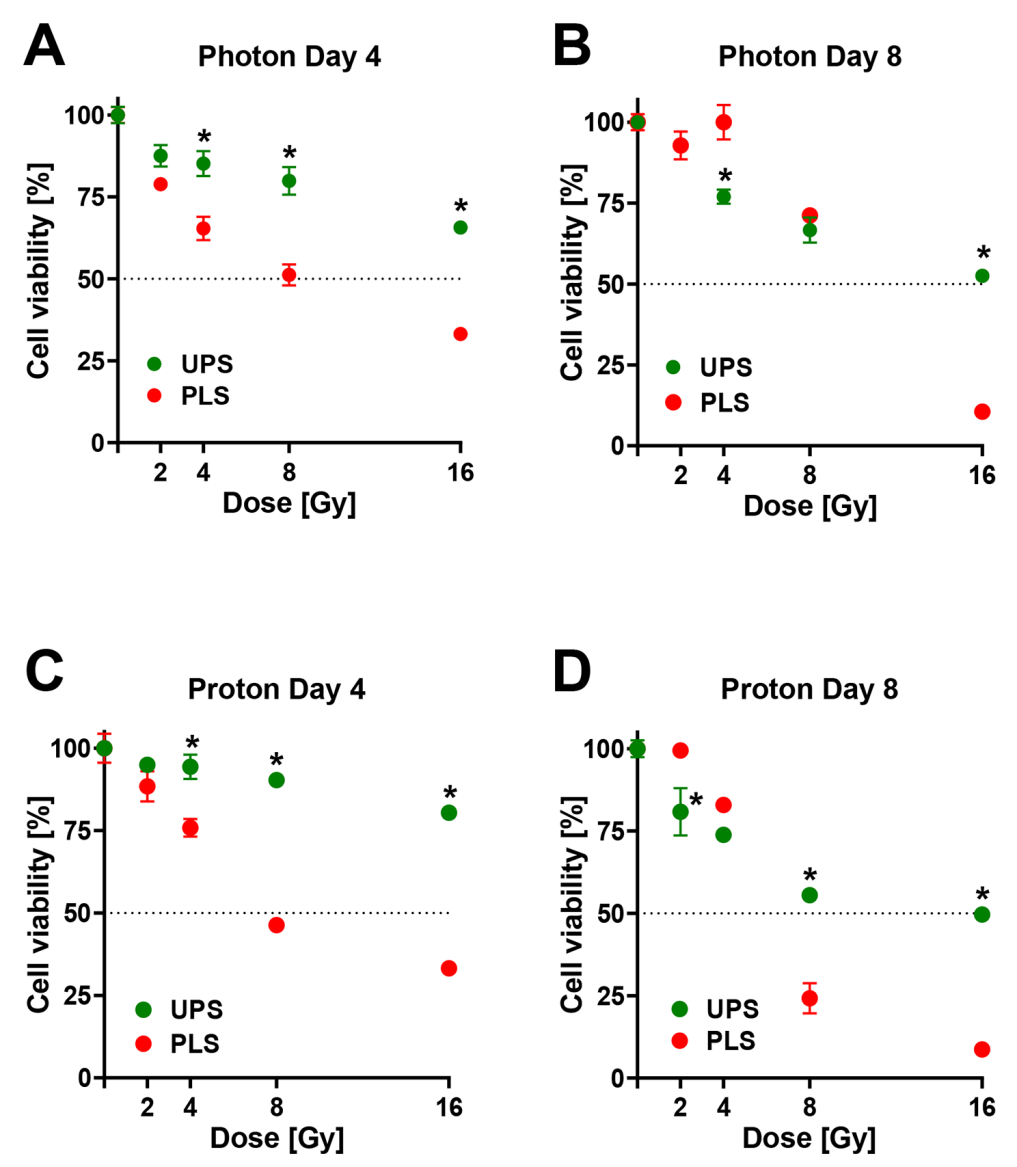
Effects of photon and proton irradiation on UPS (Sarc-P-53) vs. PLS (Sarc-P-117). Viability of UPS and PLS cell culture after four (**A, C**) and eight (**B, D**) days of incubation following increasing dosages of photon (**A, B**) or proton (**C, D**) irradiation. Mean ± standard error of the mean; *, p < 0.05 comparison between the same dosages of irradiation between UPS and PLS; at least 1 experiment with 4 technical replicates in each.

The PLS cell culture group showed significantly higher radiosensitivity towards photon and proton irradiation after four days compared to the UPS cell culture (Fig. 5A and C, p < 0.0001). Eight days after irradiation, there were no significant differences in response to photon irradiation and a significant difference after proton irradiation (Fig. 5B and D, p = 0.0979 and p = 0.0001, respectively). The PLS cell cultures were overgrown at day 8.

## Discussion

The current standard RT regimen for localized high-grade STS is far away from the idea of personalized tumor medicine. Instead, all STS subtypes receive the same preoperative RT regimen (1.8-2.0 Gy daily to a total dose of 50-50.4 Gy) despite clinical and preclinical evidence for subtype-specific differences in radiosensitivity [2–4, 6, 7, 9, 25, 44–46]. Three dimensional cell cultures have emerged as valuable preclinical models bridging the gap between animal models and humans in cancer medicine [11, 47–49]. The challenges in clinical research for STS (low incidence, high heterogeneity) are therefore met by using 3D patient-derived cell cultures to understand tumor biology and to test drug or treatment efficacy [21, 22].

Herein, we present our pilot data on cell viability after photon and proton irradiation for PD3D of high-grade STS. The UPS PD3D showed less radiosensitivity after four days in all doses and radiation modalities compared to the PLS cell culture. While in the UPS cell culture 16 Gy of photon and proton irradiation barely caused a cell-killing effect of 50% after eight days, in the PLS cell culture half of the cells were eradicated by 8 Gy (photon and proton) only four days after irradiation [6]. These findings do correlate with typical clinical outcomes for both entities. While both tumors - UPS and PLS - have mediocre clinical prognoses compared to other STS entities, UPS tumors show a higher tendency for metastases and worse survival outcomes [50–52]. Clinically, the high sensitivity of the PLS PD3D was also evident in the patient the cells were derived from. The patient received preoperative radiochemotherapy with 50.4 Gy in 28 daily fractions and surgical resection; the specimen has shown distinctly reduced proportions of viable tumor cells of 10–20%. To what extent the addition of chemotherapy has contributed to that effect cannot be assessed retrospectively. The UPS patient did not receive RT.

In both cell cultures and radiation modalities, the cell-killing effect of irradiation sustained after eight days. Although the survival measurement points in the 16 Gy dosage (photon and proton) of the UPS culture did show some degree of flattening at eight days, there was no sign of net cell number increase (Fig. 3).

The PLS cell culture on the other hand displayed a more dynamic cell turnover. After eight days, the PLS cells in the 0 Gy group overgrew in the organoid culture. Therefore, no valuable conclusions could be drawn from the results of the PLS group at eight days. Nevertheless, the PLS culture did show remarkable decreases after photon and proton irradiation at four days. This strikingly different and rather dynamic cell turnover of PLS compared to the UPS cells underlines the differences in radiosensitivity seen among both STS tumor cell cultures [6].

The physical advantages of proton irradiation are the characteristic energy deposition peak (‘Bragg peak’), behind which the energy drops towards near zero, minimizing dose deposition (“exit radiation”) in the tissue behind the Bragg peak [53]. Thereby, protons can deliver similar radiation doses to the target with 50–60% less integral or total radiation dose compared to photon intensity-modulated RT  [54–58]. These normal tissue-sparing features of proton radiation make them particularly suitable for delicate tumor locations (skull-base, spine etc.) and for pediatric patients where the risk for late toxicity or radiation-associated malignancy is the highest [53, 59–61]. Although there are no large well-matched trials comparing photon to proton therapy for sarcomas yet, many retrospective data analyses and phase II trials suggest promising low normal tissue toxicity and comparable local tumor control [59, 62–66]. In line with these findings, we only observed minimal differences in cell-killing properties between photon and proton radiation in both tumor entities at both time points (except for the PLS cell culture at eight days, which is not analyzable due to cell overgrowth). It may therefore be interesting to assess normal tissue side effects irradiation by co-culturing STS tumor cells with physiological connective tissue cells, compare viability and additionally analyze established cellular radiation-induced DNA damage response markers such as γH2AX after irradiation (photon vs. proton) at different time points [67, 68].

Our data open up a new subfield of translational sarcoma research using sarcoma PD3D to individually assess radiosensitivity in different STS entities and patients individually. The results within our own study were reproducible and stable. A potential limitation of the approach is the absence of stromal and immune cells in current 3D cell culture models, as immune cells contribute to a variety of diseases and stromal cells have become important targets in cancer drug discovery [30, 69, 70]. However, the steady evolution of such multicellular 3D tissue models with immune cells will most likely lead to new models that will be able to mimic such complex interactions [30, 71]. Another limitation is the small number of two STS PD3D used and the lack of regular re-analysis of the phenotypic stability of the PD3D. The PD3D were initially analyzed histopathologically to confirm the diagnosis of the patient they were derived from (supplementary Figs. 1 and 2). However, the analysis was not repeated to assess the maintenance of phenotopic stability as cells grew and were passaged. Moreover, certain refinements are necessary in our model such as finding the appropriate tumor cell number for the rapidly overgrowing PLS culture. Further studies with more STS entities for the 3D patient-derived sarcoma cell culture radiosensitivity assay presented herein are warranted to extent our knowledge and eventually prepare clinical-translational validation.

## Electronic supplementary material

Below is the link to the electronic supplementary material.

Supplementary Material 1

Supplementary Material 2

Supplementary Material 3

Supplementary Material 4

Supplementary Material 5

Supplementary Material 6

## Data Availability

Data available on request from the corresponding author.

## List of abbreviations

H&E: Hematoxylin and eosin
IHC: Immunohistochemistry
Ns: Not significant
PD3D: Patient-derived 3D cell culture
PLA: Polylactide
PLS: Pleomorphic liposarcoma
RT: Radiotherapy
SEM: Standard Error of the Mean
STS: Soft tissue sarcoma
UPS: Undifferentiated pleomorphic sarcoma
WHO: World Health Organization
