# Supplementary material for: Photon and Proton irradiation in Patient-derived, Three-Dimensional Soft Tissue Sarcoma Models
Source: BMC Cancer. 2023 Jun 22;23:577. doi: 10.1186/s12885-023-11013-y (PMC10286352; doi:10.1186/s12885-023-11013-y)
Supplement: Supplementary file 1 — Supplementary Material 1 [file 12885_2023_11013_MOESM1_ESM.pdf]

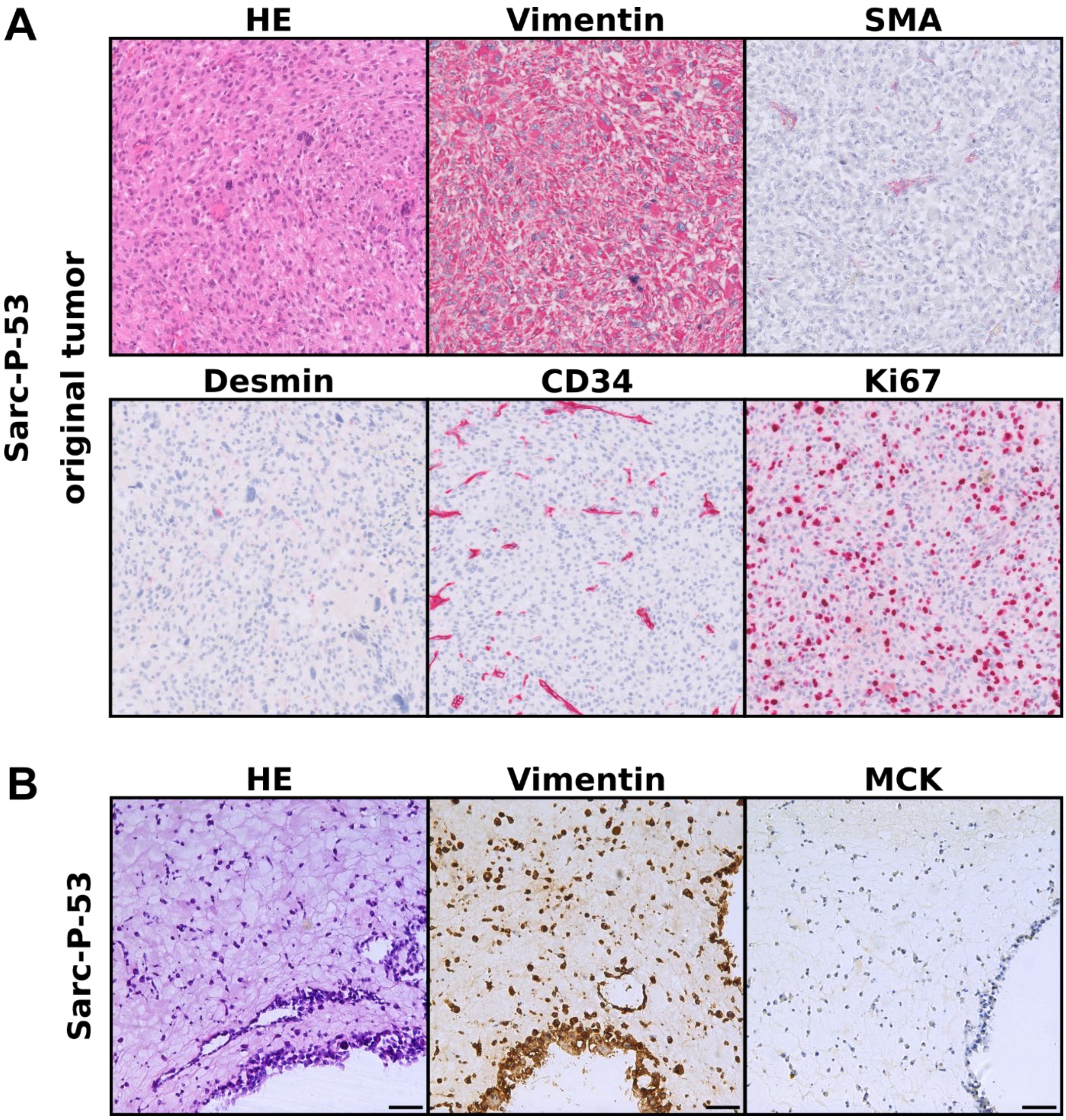

Supplementary figure 1 H&E and IHC staining from the original tumor and the PD3D model of UPS (Sarc-P-53)

(**A**) Representative H&E and IHC stainings of the original tumor, 20 x magnification. (**B**) Representative H&E and IHC stainings of the PD3D model, scale bar – 100 µm.
