# Supplementary material for: Photon and Proton irradiation in Patient-derived, Three-Dimensional Soft Tissue Sarcoma Models
Source: BMC Cancer. 2023 Jun 22;23:577. doi: 10.1186/s12885-023-11013-y (PMC10286352; doi:10.1186/s12885-023-11013-y)
Supplement: Supplementary file 3 — Supplementary Material 3 [file 12885_2023_11013_MOESM3_ESM.pdf]

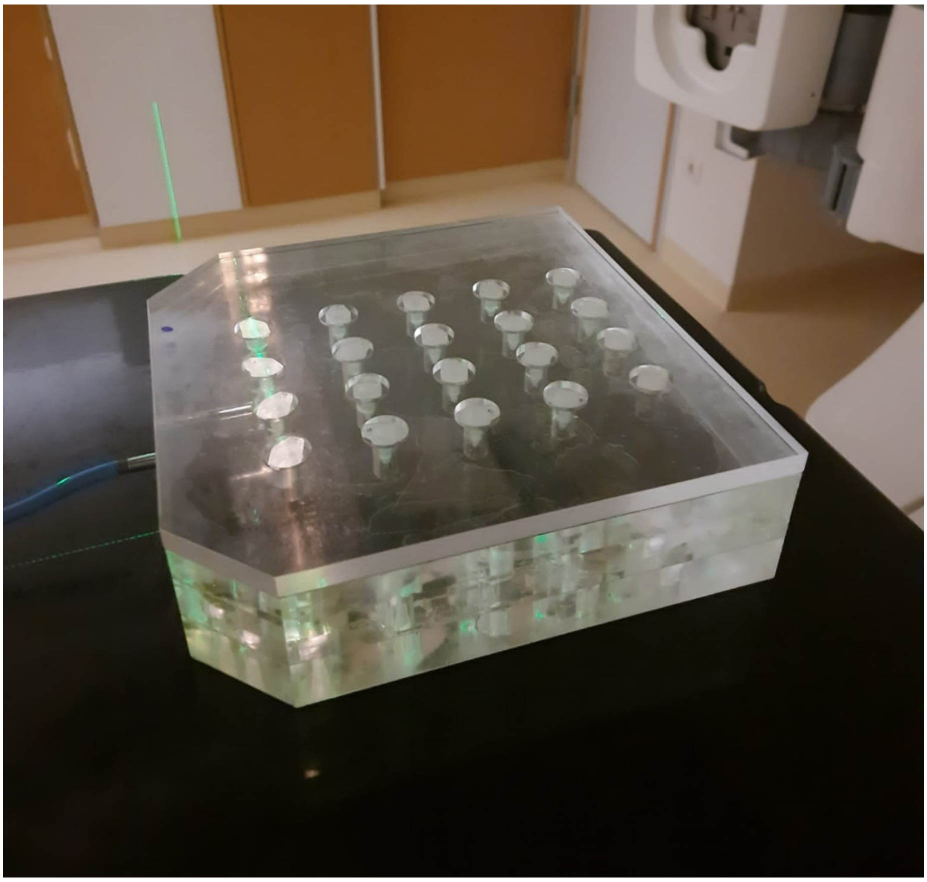

Supplementary figure 3 Plexiglass sample holder used for photon irradiation
The 1.5 ml Eppendorf tubes with samples were placed in drill holes in the plexiglass block and covered with a plexiglass top.
