# Supplementary material for: Photon and Proton irradiation in Patient-derived, Three-Dimensional Soft Tissue Sarcoma Models
Source: BMC Cancer. 2023 Jun 22;23:577. doi: 10.1186/s12885-023-11013-y (PMC10286352; doi:10.1186/s12885-023-11013-y)
Supplement: Supplementary file 4 — Supplementary Material 4 [file 12885_2023_11013_MOESM4_ESM.pdf]

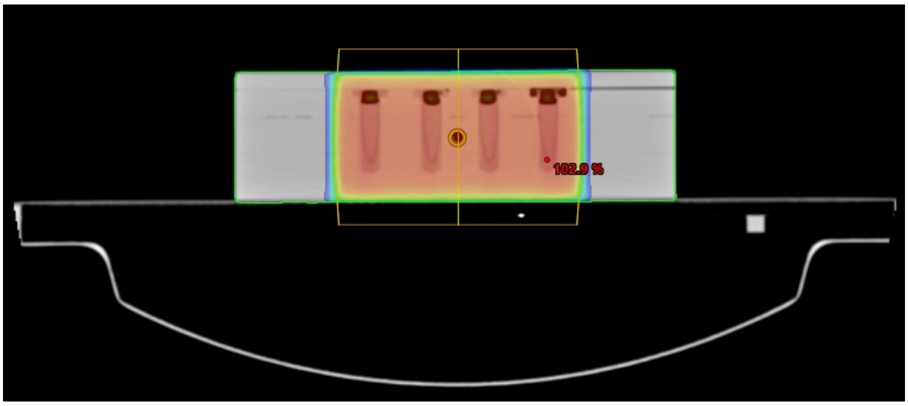

Supplementary figure 4 Computed tomography of plexiglass holder for planning of the photon irradiation

Computed tomography was used for planning of the photon irradiation of samples. The coloring indicates a homogeneous distribution of radiation in the samples.
