# Supplementary material for: Photon and Proton irradiation in Patient-derived, Three-Dimensional Soft Tissue Sarcoma Models
Source: BMC Cancer. 2023 Jun 22;23:577. doi: 10.1186/s12885-023-11013-y (PMC10286352; doi:10.1186/s12885-023-11013-y)
Supplement: Supplementary file 5 — Supplementary Material 5 [file 12885_2023_11013_MOESM5_ESM.pdf]

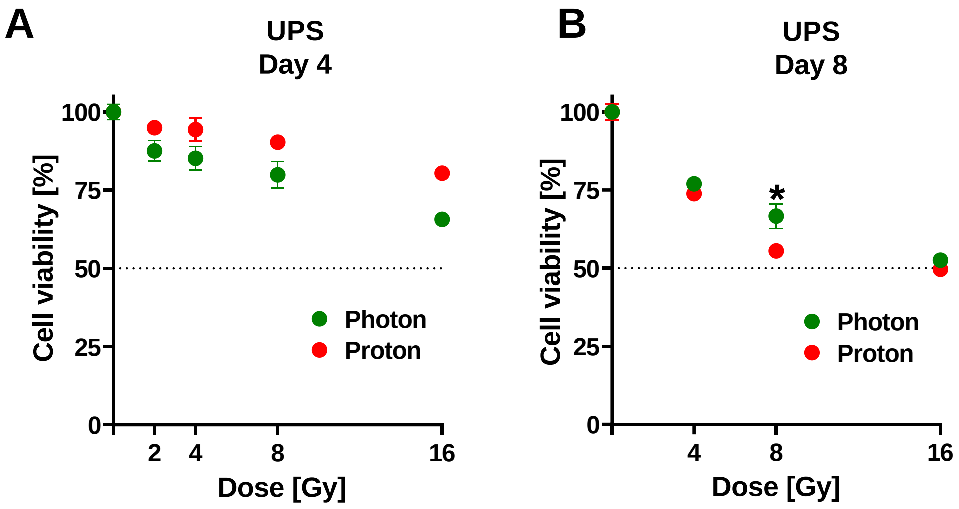

Supplementary figure 5 Effects of photon vs. proton irradiation on UPS (Sarc-P-53)

Viability of UPS cell culture after 4 (**A**) and 8 (**B**) days of incubation following increasing dosages of photon and proton irradiation. Mean ± standard error of the mean; *, $p<0.05$ comparison between the same dosages of photon vs. proton irradiation; at least one experiment with 4 technical replicates in each.
