# Supplementary material for: Photon and Proton irradiation in Patient-derived, Three-Dimensional Soft Tissue Sarcoma Models
Source: BMC Cancer. 2023 Jun 22;23:577. doi: 10.1186/s12885-023-11013-y (PMC10286352; doi:10.1186/s12885-023-11013-y)
Supplement: Supplementary file 6 — Supplementary Material 6 [file 12885_2023_11013_MOESM6_ESM.pdf]

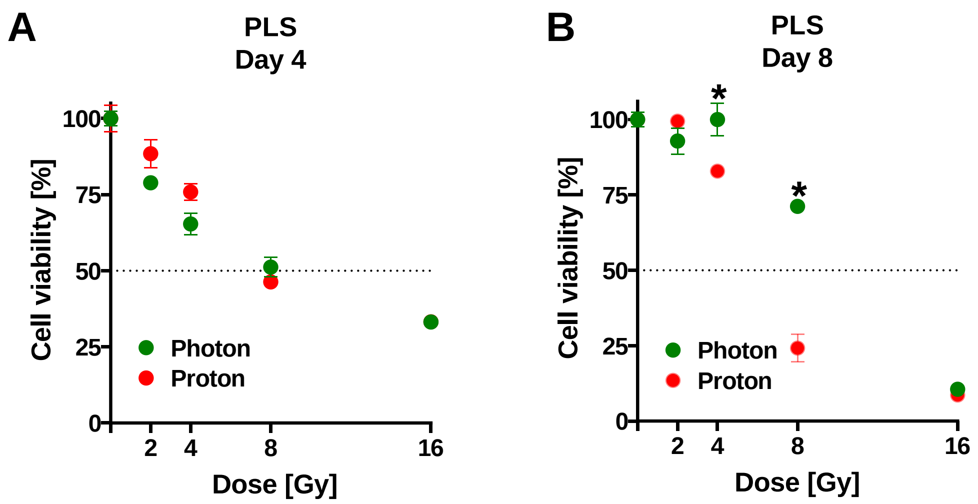

Supplementary figure 6 Effects of photon vs. proton irradiation on PLS (Sarc-P-117)

Viability of PLS cell culture after 4 (A) and 8 (B) days of incubation following increasing dosages of photon and proton irradiation. Mean ± standard error of the mean; empty circles, cells overgrown; *, p<0.05 comparison between the same dosages of photon vs. proton irradiation; at least one experiment with 4 technical replicates in each.
